# Onion (*Allium cepa* L.)-Derived Nanoparticles Inhibited LPS-Induced Nitrate Production, However, Their Intracellular Incorporation by Endocytosis Was Not Involved in This Effect on RAW264 Cells

**DOI:** 10.3390/molecules26092763

**Published:** 2021-05-07

**Authors:** Masao Yamasaki, Yumi Yamasaki, Rina Furusho, Hayaka Kimura, Ichiro Kamei, Hiroko Sonoda, Masahiro Ikeda, Tatsuya Oshima, Kenjiro Ogawa, Kazuo Nishiyama

**Affiliations:** 1 Department of Biochemistry and Applied Biosciences, University of Miyazaki, 1-1 Gakuenkibanadai-nishi, Miyazaki 889-2192, Japan; shopin_piano@yahoo.co.jp (R.F.); nishiyam@cc.miyazaki-u.ac.jp (K.N.); 2 Faculty of Regional Innovation, University of Miyazaki, 1-1 Gakuenkibanadai-nishi, Miyazaki 889-2192, Japan; yamasakiy@cc.miyazaki-u.ac.jp (Y.Y.); habo26092@gmail.com (H.K.); 3 Department of Forest and Environmental Sciences, University of Miyazaki, 1-1 Gakuenkibanadai-nishi, Miyazaki 889-2192, Japan; kamei@cc.miyazaki-u.ac.jp; 4 Department of Veterinary Science, University of Miyazaki, 1-1 Gakuenkibanadai-nishi, Miyazaki 889-2192, Japan; sonoda-h@cc.miyazaki-u.ac.jp (H.S.); a0d302u@cc.miyazaki-u.ac.jp (M.I.); 5 Department of Applied Chemistry, University of Miyazaki, 1-1 Gakuenkibanadai-nishi, Miyazaki 889-2192, Japan; oshimat@cc.miyazaki-u.ac.jp; 6 Organization for Promotion of Tenure Track, University of Miyazaki, 1-1 Gakuenkibanadai-nishi, Miyazaki 889-2192, Japan; ogawa.kenjirou.u2@cc.miyazaki-u.ac.jp

**Keywords:** *Allium cepa* L., nanovesicles, lipopolysaccharide, endocytosis

## Abstract

The aim of this study was to evaluate the involvement of nanoparticles prepared from *Allium cepa* L. as anti-inflammatory agents. In the present study, we identified nanoparticles from *Allium cepa* L. using the ultracentrifugation exosome purification method. The nanoparticles were referred to as 17,000× *g* and 200,000× *g* precipitates, and they contained quercetins, proteins, lipids, and small-sized RNA. The nanoparticles inhibited nitric oxide production from lipopolysaccharide (LPS)-stimulated RAW264 cells without cytotoxic properties. Cellular incorporation was confirmed by laser microscopic observation after PKH26 staining. The inhibition of caveolae-dependent endocytosis and macropinocytosis significantly prevented the incorporation of the nanoparticles but had no effect on the inhibition of nitric oxide in RAW264 cells. Collectively, the identified nanoparticles were capable of inhibiting the LPS response via extracellular mechanisms. Taken together, the way of consuming *Allium cepa* L. without collapsing the nanoparticles is expected to provide an efficient anti-inflammatory effect.

## 1. Introduction

The health-promoting effects of natural plant resources, especially vegetables and fruits, have attracted attention not only from researchers but also among general consumers. Numerous studies have focused on the identification and purification of active components in food, and these studies have certainly contributed to the development of functional foods. Although these studies enabled depicting the detailed mechanisms of food function at the molecular level, we are interested in the difference between unpurified or unprocessed foods and purified compounds in terms of their health-promoting effects. Actually, accumulated evidence indicates why eating raw and unprocessed foods should be prioritized from the point of view of the prevention of various diseases [1,2]. One intriguing explanation of this is eating organellas such as mitochondria, chloroplast and vacuoles in the case of raw and unprocessed food [3,4,5]. Therefore, the ingestion of plant cell structures may at least partly explain the health benefit of consuming raw and unprocessed foods. In this context, exosome-like nanoparticles found in various vegetables and fruit have health-promoting effects. These nanoparticles are carriers for microRNAs, proteins, lipids, and vitamins [6,7,8,9,10,11]. The encapsulation of these compounds might confer protection against degradation and further metabolism, and the delivery of these nanoparticles to the target tissues or cells might manipulate cellular function [5]. For instance, ginger-derived nanoparticles, but not those found in grapes, grapefruits or carrots, strongly induce anti-inflammatory cytokines, suggesting differences in the physiological properties of these species [6]. Interestingly, extracellular vesicles can be found even in dried and heated plants [10,11], presenting an attractive and novel approach for the evaluation of food function not only in fresh plants but also appropriate food processing. Onion (*Allium cepa* L.) is one of the most popular vegetables in the world and is well known for its health benefits. It is rich in flavonoids, such as quercetin and its glucosides, sulfuric compounds that are responsible for the physiological function of onion [12,13]. Among its physiological functions, anti-inflammation is one of the most interesting; the anti-inflammatory property suggests that these components can prevent and alleviate inflammatory-related disorders, such as allergies and lifestyle diseases [14,15]. A previous in vitro study also revealed that onion peel extract inhibits the lipopolysaccharide (LPS)-induced response in human colon cancer cells [16], and crude onion powder inhibits the receptor activator of nuclear factor kappa-B ligand (RANKL)-induced nuclear factor kappa-B (NF-κB) activation in RAW264.7 cells [17]. Based on previous studies, it can be postulated that nanoparticles are ubiquitously found in vegetables and fruits. In this study, we hypothesized that nanoparticles can be prepared from onions, and these particles have anti-inflammatory properties. 

The nanoparticles are likely to collapse and disappear during excess food processing. The aim of this study was to reveal the efficiency of the anti-inflammatory effect derived from onion nanoparticles to propose an advantage of eating onions without excessive food-processing. Specifically, we purified the nanoparticles from onions according to the exosome preparation method to evaluate their anti-inflammatory properties and cellular uptake in LPS-stimulated RAW264 cells.

## 2. Results

### 2.1. Profiles of 17 Kp and 200 Kp

**Figure 1:**
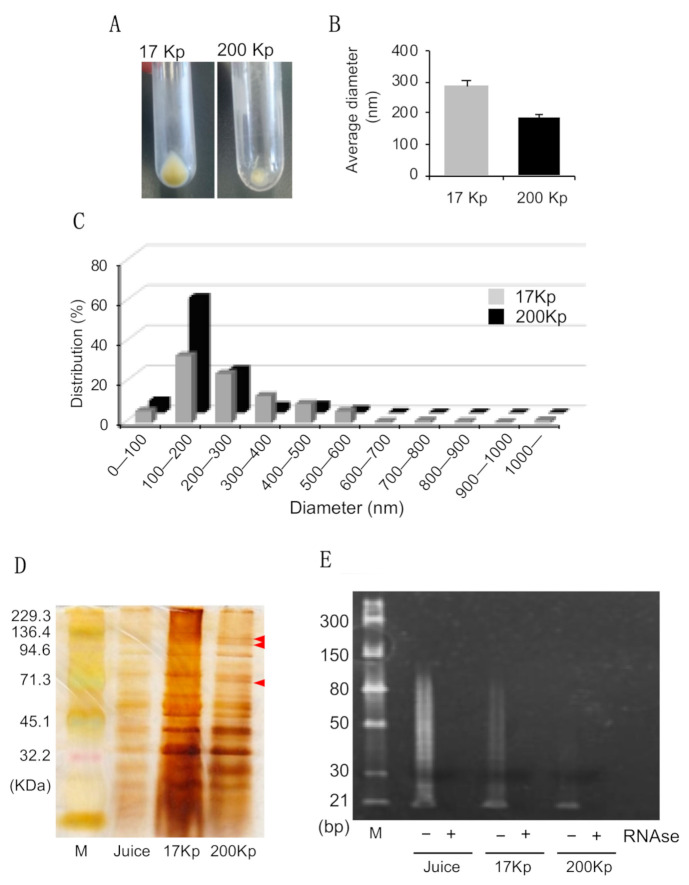
Profiles of nanoparticles identified from *Allium cepa* L. Nanoparticles were identified as 17,000× *g* and 200,000× *g* precipitates, referred to as 17 Kp and 200 Kp, respectively. (**A**): Appearance of 17 Kp and 200 Kp; (**B**,**C**): Diameters of 17 Kp and 200 Kp were measured by dynamic light scattering method. B shows the average diameter and C shows the distribution of the diameter. (**D**): Protein profiles analyzed by SDS-PAGE and silver staining. Red arrows show specific bands found in 200 Kp. (**E**): Small RNA profiles analyzed by PAGE. Small RNA purified from 17 Kp and 200 Kp were treated with or without RNAseA before loading to the electrophoresis.

After centrifugation at 17,000× *g* and 200,000× *g*, apparent pellets with a faint yellow color were observed, and we referred to them as 17 Kp and 200 Kp, respectively.

(Figure 1A). These pellets were suspended in the phosphate buffered saline and passed through the 0.45 μm membrane filter to evaluate their diameters of 17 Kp and 200 Kp. The diameters of 17 Kp and 200 Kp could not be measured without membrane filtration. The average diameters of 17 Kp and 200 Kp were 288.1 nm and 185.3 nm, respectively, and the size distribution data showed that more than 80% of 200 Kp was less than 300 nm.

(Figure 1B,C). To estimate the content of 17 Kp and 200 Kp, proteins and small-sized RNA were extracted and detected by electrophoresis. SDS-PAGE and silver-staining data showed that 17 Kp and 200 Kp contained proteins; although the protein profile was similar between onion juice of 17 Kp and 200 Kp, some bands were specifically detected at 200 Kp, highlighted as red arrows (Figure 1D). Figure 1E shows the electrophoresis image of the small RNA analysis. Several previous reports revealed that nanoparticles derived from edible plants included small RNA, such as miRNAs; we purified small RNA from the total RNA extract, and RNaseA treatment was performed to verify the RNA detection. Data showed that both 17 Kp and 200 Kp included small (20~80 bp)-sized RNA.

(Figure 1E). Then, we measured representative flavonoids in onion quercetin, Q4′G, and Q3,4′G. Here, 48 mL juice was prepared from two onions for the preparation of 17 Kp and 200 Kp, therefore, their juice contains 17 Kp and 200 Kp. Quercetins were extracted by methanol then quercetins content in 48 mL juice as encapsulated in 17 Kp and 200 Kp were measured and data were shown as μg/mL juice and % of juice (Table 1). Most of the quercetins occurred as glucosides in juice, 17 Kp and 200 Kp. Here, juice was a liquid before 17,000× *g* centrifugation, therefore, included 17 Kp and 200 Kp. Total quercetin (quercetin + Q4′G + Q3,4′G) concentrations in juice, 17 Kp and 200 Kp were 171.5, 7.7 and 3.8 μg/mL juice; therefore, 4.5% and 2.2% of total quercetins in juice were estimated to be encapsulated in 17 Kp and 200 Kp. Q3G and other flavonoids were not detected in any of the samples studied.

### 2.2. Effect of 17 Kp and 200 Kp on LPS Response

**Figure 2:**
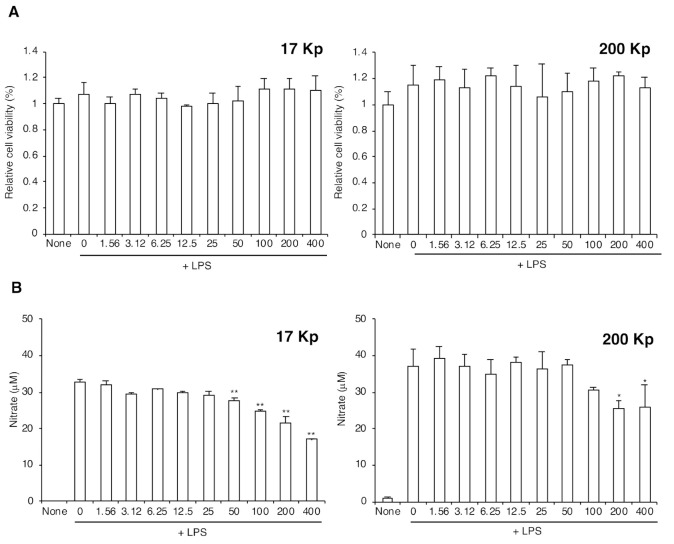
Effect of 17 Kp and 200 Kp on the nitric oxide production in lipopolysaccharide (LPS)-treated RAW264 cells. Cell were treated with 17 Kp or 200 Kp for 24 h then treated with or without LPS (100 ng/mL) for 24 h. (**A**): Cytotoxicity was evaluated by using Cell Counting Kit-8, and data are shown as relative cell viability (data in none were shown as 1). (**B**): Nitric oxide was detected as nitrate by Griess method. Data are means ± SE for 3 experiments. Asterisk indicates significant difference from the 0 + LPS group at * p < 0.05, ** *p <* 0.01.

To evaluate the physiological function, the effect of 17 Kp and 200 Kp on NO production from LPS-treated RAW264 cells was measured. Cells were treated with 17 Kp and 200 Kp for 24 h, and then treated with 100 ng/ mL of LPS for 24 h. Cell viability was also examined, but no cytotoxic effect of 17 Kp and 200 Kp was observed at any given concentrations (Figure 2A). LPS drastically increased NO production, whereas both 17 Kp and 200 Kp significantly inhibited it at 50–400 and 100–400 μg protein/mL, respectively.

(Figure 2B). The precise number or weight of the nanoparticles cannot be measured; therefore, we expressed the amount of 17 Kp and 200 Kp as concentrations of proteins. The 17 Kp and 200 Kp had no effect on the NO production without LPS treatment (data not shown).

### 2.3. Intracellular Incorporation of 17 Kp and 200 Kp

**Figure 3:**
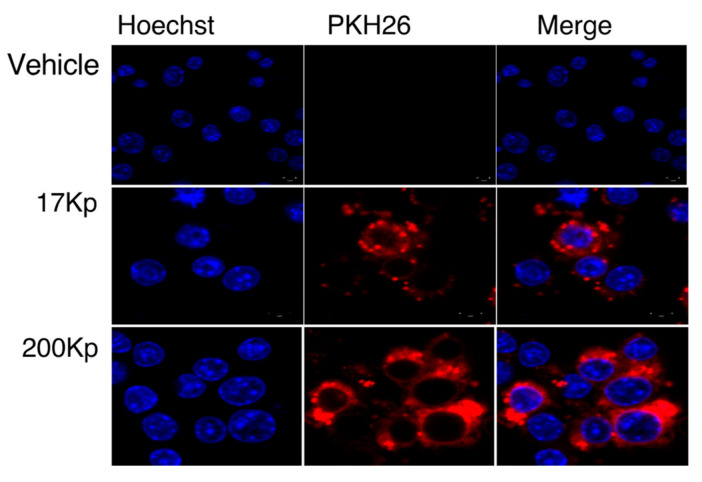
Intracellular incorporation of 17 Kp and 200 Kp into RAW264 cells. The 17 Kp or 200 Kp were stained with PKH26. Cells were treated with 400 μg protein/mL 17 Kp or 200 Kp for 2 h. Then, the nuclei were stained with Hoechst33342. Cells were observed by confocal laser microscope.

We then evaluated the incorporation of 17 Kp and 200 Kp into RAW264 cells. Here, we found that 17 Kp and 200 Kp were well stained with PKH26, which is a red fluorescent dye used for staining lipophilic membranes and extracellular vesicles [14]. To verify the occurrence of lipids, we extracted lipids by a chloroform: methanol (2:1) solution from the 17 Kp and 200 Kp samples and found that they included 313.7 and 150.2 mg lipids/mg protein, respectively. PKH26-stained 17 Kp and 200 Kp were clearly observed in the intracellular regions of RAW264 cells after 2 h of treatment (Figure 3).

### 2.4. Effect Endocytosis Inhibitors on PKH26 Stained 17 Kp and 200 Kp Uptake

**Figure 4:**
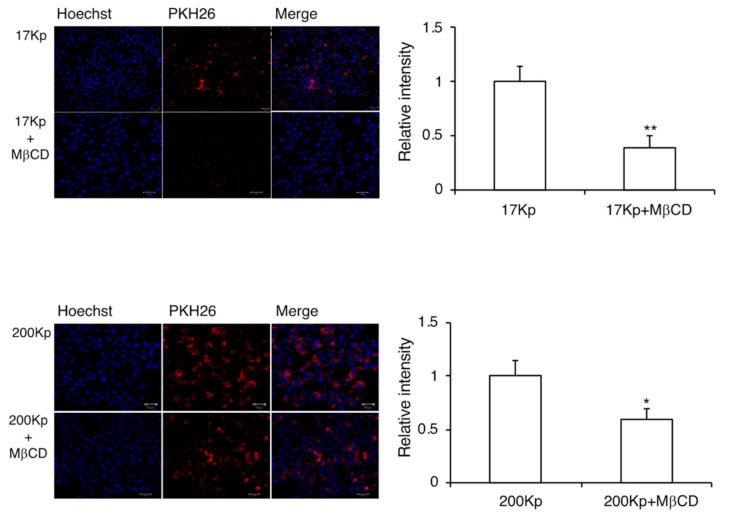
Effect of the clathrin-dependent endocytosis inhibitor on the intracellular incorporation of 17 Kp and 200 Kp into RAW264 cells. The 17 Kp or 200 Kp were stained with PKH26. Cells were treated with 10 mM of MβCD for 1 h, then 400 μg protein/mL 17 Kp or 200 Kp for 2 h. Nuclei were stained with Hoechst33342. Cells were observed by confocal laser microscope. Asterisk indicates significant difference from the 17Kp or 200Kp group at * p < 0.05, ** *p <* 0.01.

**Figure 5:**
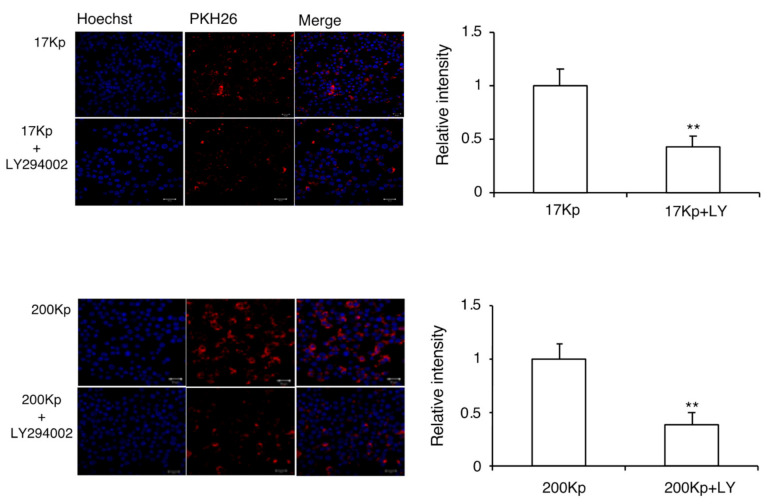
Effect of the macropinocytosis inhibitor on the intracellular incorporation of 17 Kp and 200 Kp into RAW264 cells. The 17 Kp or 200 Kp were stained with PKH26. Cells were treated with 50 μM of LY294002 for 1 h, then 400 μg protein/mL 17 Kp or 200 Kp for 2 h. Nuclei were stained with Hoechst33342. Cells were observed by confocal laser microscope. Asterisk indicates significant difference from the 17Kp or 200Kp group at ** *p <* 0.01.

**Figure 6:**
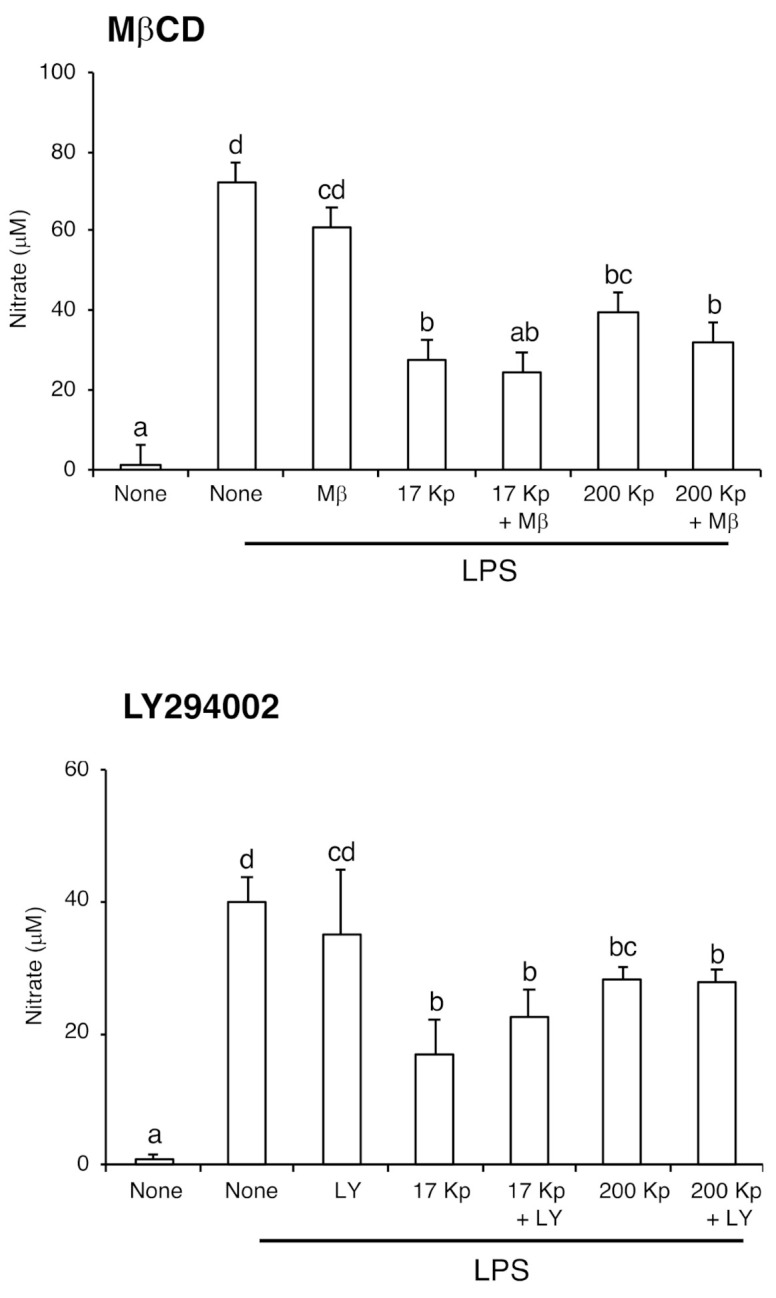
Effect of endocytosis inhibitors on the nitric oxide production in lipopolysaccharide (LPS)-treated RAW264 cells. Cell were treated with 400 μg protein/mL of 17 Kp or 200 Kp for 24 h, then treated with or without inhibitors for 1 h. Finally, cells were treated with LPS (100 ng/mL) for 24 h. Nitric oxide was detected as nitrate by Griess method. Data are shown as mean ± SE for 3 experiments. Data without any common alphabetic letter are significantly different each other at *p* < 0.05.

To clarify the intracellular uptake mechanism of 17 Kp and 200 Kp, the cells were co-treated with MβCD (inhibitor for caveolae-dependent endocytosis) or LY294002 (inhibitor for macropinocytosis) (Figure 4 and Figure 5). The fluorescence intensity of PKH26 was quantified for the cellular uptake of 17 Kp and 200 Kp. Interestingly, both MβCD and LY294002 incompletely but significantly prevented the uptake of 17 Kp and 200 Kp. Chlorpromazine, an inhibitor for clathrin-dependent endocytosis, failed to prevent the uptake of 17 Kp and 200 Kp (data not shown). Based on these results, we examined the effect of these inhibitors on the function of 17 Kp and 200 Kp. Unexpectedly, MβCD and LY294 did not affect the 17 Kp- and 200 Kp-induced inhibition of NO production in LPS-treated cells (Figure 6).

## 3. Discussion

In the present study, we evaluated the anti-inflammatory properties of onion-derived nanoparticles on RAW264 cells. First, we succeeded in preparing 17 Kp and 200 Kp according to the method used for the purification of exosomes [18,19]. In addition, dynamic light scattering analysis and electrophoresis data showed that both 17 Kp and 200 Kp were similar to the exosome, in that they were nano-sized and included proteins and small sized RNA (Figure 1). Here, we did not measure the zeta potential that enables the prediction of aggregation. We performed 0.45 μm filtration to eliminate the large size of particles that strongly influence the dynamic light scattering analysis even if the nanoparticle content is extremely low. Actually, without filtering we could not acquire the data indicating the occurrence of a large particle. As for HPLC and cell culture experiments, we quickly applied for the experiment just after the thawing; on the other hand, it took a few hours after thawing for dynamic light scattering analysis to indicate that partial aggregation might occur in a few hours’ time. Presently, we did not identify the specific proteins in 200 Kp, but these proteins are likely molecular markers for the nanovesicles, such as CD9, CD63, and CD81, in the exosome [20]. Several previous studies, in which nanoparticles were purified from various edible plants, showed that miRNA was present in the nanoparticles [21,22]. This information implies that an edible plant derived might be vectors of small RNA to predator cells result in the regulation of the gene expression. Artificial nanoparticles and animal-derived exosomes are being attracted as putative materials for drug delivery [23] and edible plant-derived nanoparticles containing plant-derived miRNAs are also emerging candidates for the therapeutics of various diseases [24,25]. Of note, plant RNAs are capable of modulating immune function such as the regulation of response to TLR-4 stimulation in dendritic cells [26]. Our data also showed that 17 Kp and 200 Kp included small-sized (20~80 bp) RNA; therefore, small-sized RNA, such as miRNAs, might modulate the gene expression in cells treated with 17 Kp and 200 Kp. Several miRNAs have been identified in the onion [27].

It has been reported that onion juice and peel extract-attenuated LPS response in colon carcinoma and LPS-induced osteoclastogenesis in RAW264.7 cells [16,17]. Our preliminary data also showed that onion juice suppressed LPS-induced NO production in RAW264 cells, verifying the inhibition of the LPS response. The onion contains several polyphenols, and quercetin and its glucoside can exert health benefits. Quercetin was partly responsible for the inhibition of the LPS response in RANKL/LPS-induced osteoclastogenesis in RAW264.7 cells [28]. The onion juice used in this study contained 4.9, 68.6, and 98.0 μg/mL of quercetin, Q-4′G, and Q-3, 4′G, respectively. This is similar to previous reports [29,30]. The total quercetin (quercetin + Q-4′G + Q-3, 4′G) concentration in juice, including 17 Kp and 200 Kp, was 171.5 μg/mL. Table 1 shows that 17 Kp and 200 Kp also contained 7.7 and 3.8 μg/mL, representing 4.5 and 2.2% of quercetins, respectively, of these flavonoids, suggesting that they are polyphenol-inclusive vesicles. Both particles were faint yellow in appearance. Quercetins are yellow pigmented and form in vacuoles in the soluble sugar-binding form in the plant cells; therefore, 17 Kp and 200 Kp might contain vacuole-derived intracellular or extracellular particles.

Figure 2 shows that 17 Kp and 200 Kp inhibited the production of NO in a dose-dependent manner. The sample concentrations are shown as protein levels, because we could not precisely quantify the number and weight of the samples. For instance, 400 μg/mL of protein corresponded to 581 and 260 ng/mL (1.1 μM and 0.48 μM, respectively) of the total quercetins. Significant effects were observed from both 17 Kp and 200 Kp at a concentration of 200 μg/mL. Moreover, 17 Kp showed a significant effect at 50 μg/mL (137.5 nM). It is possible that these concentrations are too low to exert an inhibitory effect on LPS response. In addition, none of the quercetins were detected in cells treated with 17 Kp and 200 Kp. It was considered that at 17 Kp and 200 Kp, flavonoid capsules occur—at least in flesh juice. However, quercetins encapsulated in 17 Kp and 200 Kp were not involved in the anti-inflammatory properties or further applications, quercetin nanoparticles are gaining attention as potent cancer therapeutic materials [31]. 

The exosome is an extracellular nanoparticle ubiquitously released from cells in animal tissues and organs. Exosomes include proteins, lipids, mRNA, and miRNA, and are absorbed by target cells. This results in a change in mRNA expression and the cellular phenotype. In this study, we hypothesized that 17 Kp and 200 Kp exert their effects by their cellular uptake via an endocytosis approach [32]. Confocal laser microscope images indicated that PKH26-stained 17 Kp and 200 Kp were absorbed by RAW264 cells, and this absorption was prevented by MβCD or LY294002 treatment. These data indicated that 17 Kp and 200 Kp were absorbed via a caveolae-mediated endocytosis and macropinocytosis. One possibility was that cellular uptake was indispensable for the effect on RAW264 cells; however, endocytosis inhibitors failed to prevent the inhibitory effect of 17 Kp and 200 Kp on NO production. Another possibility is that both endocytotic pathways synergistically contribute to the effect of 17 Kp and 200 Kp. Confocal microscope observation clearly showed the intracellular incorporation of 17 Kp and 200 Kp, although quercetin and its glucosides were not detected from cell lysates. These data imply a low efficiency of cellular incorporation of 17 Kp and 200 Kp. At this point, both 17 Kp and 200 Kp must not be homogenous in terms of their physical properties and chemical components; therefore, specific particles may be efficiently incorporated and be responsible for the effect on RAW264 cells.

Previous reports, in which nanoparticles were purified from edible plants, showed that nanoparticles were structured like lipid membranes and contained phospholipids; however, their profiles were distinct from each other [24]. Although we also confirmed that 17 Kp and 200 Kp contained 313.7 and 150.2 mg lipids/mg protein, respectively, the extracted lipid treatment of RAW264 failed to prevent the LPS response (data not shown). This study could not completely repudiate the relationship between lipid involvement and the function of 17 Kp and 200 Kp. Further studies are needed to clarify the molecular mechanism of 17 Kp and 200 Kp on RAW264 cells and to evaluate the additional beneficial effects of 17 Kp and 200 Kp.

At first, we have shown that the anti-inflammatory effect of nanoparticle from *Allium cepa* L. It is estimated that the structure and content of nanoparticle might be collapsed or denatured during food processing such as heating, drying, high pressure and extraction. Finally, we propose the advantage of eating *Allium cepa* L. without denaturing the nanoparticles to accomplish its potent anti-inflammatory activity.

## 4. Materials and Methods

### 4.1. Preparation of Nanovesicles

*Allium cepa* L. was purchased from a commercial supermarket in Miyazaki city and mashed with a food processor (MK-K48-W, National, Osaka, Japan); then, juice was obtained using a squeezer. Juice was recovered into 50 mL polycarbonate tubes and centrifuged at 1000× *g* for 5 min at room temperature to remove the residue. Supernatant was further centrifuged at 17,000× *g* for 15 min, and its precipitates are referred to as 17 Kp throughout this manuscript. The supernatant was further ultracentrifuged at 200,000× *g* for 1 h, and those precipitates are referred to as 200 Kp throughout this manuscript. Samples were reserved at −80 °C before analysis. Diameters of the nanovesicles were measured by dynamic light scattering method using a SZ-100 nanoPartica series instrument (Horiba, Ltd., Kyoto, Japan).

### 4.2. Small RNA Extraction and Purification

Extraction of small RNA was performed using the miRNeasy Mini kit (Qiagen, Hilden, Germany) according to the manufacture’s protocol. At the end of the procedure, 40 μL of RNAase-free water was added to the spin column and centrifuged at 8000× *g* for 1 min. The eluted solution was diluted 200-fold with RNAase-free water and analyzed by the following electrophoresis protocol. To verify the detection of RNA, samples were treated with 10 μg/mL of RNAseA (Sigma, St. Louis, MO, USA) at 37 °C for 30 min. Samples were separated by electrophoresis on a 15% SuperSep^TM^Ace polyacrylamide gel (Fujifilm Wako Pure Chemical Co., Osaka, Japan) and stained with ethidium bromide.

### 4.3. Protein Analysis

The preparation of cellular protein samples was performed according to our previous method [33]. The 17 Kp and 200 Kp samples were treated with lysate buffer (50 mM Tris-HCl, 150 mM NaCl, 2% Triton-X 100, 1 mM EDTA, 50 mM NaF, 30 mM Na_4_P_2_O_7_) including the 1/50 vol. protease inhibitor cocktail (Nacalai, Kyoto, Japan). Protein concentrations were measured using the BCA protein assay reagent (Pierce, Rockford, IL, USA). Lysates containing 10 μg of protein were separated by electrophoresis on a 10% SDS-polyacrylamide gel. Proteins were stained with 2D-Silver Stain Reagent II according to the appended protocol (Cosmo Bio, Tokyo, Japan).

### 4.4. High-Performance Liquid Chromatography (HPLC) Analysis for the Detection of Quercetins

Quercetins were quantified by using HPLC according to a previous report with some modifications [34]. To extract the flavonoids, 200 μL of 50% methanol was added to 17 Kp and 200 Kp and sonicated. The solution was passed through a 0.45 μm syringe filter (Merck, Darmstadt, Germany) before analysis. Quercetin and its glucosides, quercetin-4′-glucoside (Q4′G) and quercetin-3,4′-glucoside (Q3,4′G), were measured by HPLC equipped with a PDA detector system at 360 nm (Shimadzu, Kyoto, Japan) using the ODS-2 column (GL Science, Tokyo, Japan) at 30 ℃. The mobile phase consisted of methanol (A) and 0.5% formic acid (B), and the employing gradient (0–5 min, A/B 5% 5–15 min A/B 20%, 15–20 min A/B 30%, 20–30 min A/B 50%, 30–35 min A/B 100%) flowed at 1.0 mL/min. Accurately weighed quercetin (Cayman Chemical, MI, USA), Q4′G, and Q3,4′G (Tokiwa Phytochmical, Chiba, Japan) were dissolved in methanol to prepare the 1 mg/mL stock solution and kept at −30 °C before analysis. Quercetins in the samples were identified by comparison with the retention times of these standards. 

### 4.5. Measurement of Nitric Oxide (NO) by RAW264 Cells

RAW264 cells were purchased from Riken Cell Bank (RCB0535, Tsukuba, Japan). Cells were cultured in Dulbecco’s modified Eagle medium (DMEM) supplemented with 10% fetal bovine serum (Sigma, MO, USA) and 1/100 vol. penicillin–streptomycin–amphotericin B cocktail (Fujifilm Wako). Cells (4th passage) were seeded at 1.8 × 10^4^ cells/cm^2^ in a 90 mm dish, and then maintained every second day to make the cells at the growth state. Experiments were done using cells within 24 passages after thawing. Cells were seeded at 5.0 × 10^4^ cells/cm^2^ and preculture for 24 h before analysis to reach to subconfluent (approximately 70%). Then, the cells were treated with given concentrations of the samples in phenol red free DMEM for 24 h; then, LPS (Sigma L6511) was added at 100 ng/mL. After 24 h culture, cultured supernatant was recovered to measure the production of NO from RAW264 cells. NO production was measured as nitrate using the Griess method as the previous report [33]. Briefly, the supernatant was mixed with Griess reagent, 2.5% phosphoric acid, 1% sulfanilamide, and 0.1% *N*-(1-Naphthyl) ethylene-diamine dihydrochloride, and absorbance was measured at 540 nm. Cytotoxicity of the samples were measured using Cell Counting Kit-8 (Dojindo, Kumamoto, Japan).

### 4.6. Staining and Observation of Nanovesicles

Nanoparticles were stained with PKH26, as referred to in a previous report [35]. The 17 Kp and 200 Kp samples were suspended in 1.2 mL phosphate buffered saline and mixed with 600 μL of PKH26 staining solution. This mixture was incubated at 37 °C for 30 min. Staining reaction was stopped by the addition of 1.8 mL of 1% bovine serum albumin. Then, the suspension was ultracentrifuged at 200,000× *g* for 1 h to recover the stained nanoparticles. To evaluate the cellular incorporation of 17 Kp and 200 Kp, RAW264 cells were inoculated into 35 mm glass bottom dish (Iwaki 3911-035, Shizuoka, Japan). RAW264 cells were treated with PKH26-stained 17 Kp and 200 Kp for 2 h, then nuclei were stained with 10 μg/mL Hoechst33342. Cells were mounted with 50% glycerol and observed by confocal laser microscope LSM700 (Carl Zeiss, Oberkochen, Germany). The intensity of PKH26 red fluorescent was quantified by Leica Application Suite X software to evaluate the intensity of cellular incorporation. Ten micromolar of MβCD (inhibitor for caveolae-dependent endocytosis) or 50 μM of LY294002 (inhibitor for macropinocytosis) were added 1 h before the 17 Kp and 200 Kp treatments.

### 4.7. Statistical Analysis

Statistical differences among experimental groups were evaluated by Student’s *t*-test or Duncan’s multiple range test (statistical analysis for Mac Ver.3.0, Esumi, Tokyo, Japan). The statistical significance was defined as *p* < 0.05.

## Figures and Tables

**Table 1:** Content of quercetins in onion juice, 17 Kp and 200 Kp.

μg/mL Juice	Juice	17 Kp	200 Kp
Quercetin	4.92 ± 2.00	0.15 ± 0.05	0.12 ± 0.03
Q4′G	68.6 ± 16.0 ^a^	4.68 ± 1.85 ^b^	1.61 ± 0.44 ^b^
Q3,4′G	98.0 ± 19.7 ^a^	2.85 ± 0.77 ^b^	2.07 ± 0.58 ^b^

Data are means ± SE for 3 measurements. Data without any common alphabetic letter are significantly different each other at *p* < 0.05.

## Data Availability

Data contained within this article is available from the authors.

## Sample Availability

Samples are available from the authors.
